# 2-[(4-Chloro­benz­yl)sulfanyl]-4-(2-methyl­prop­yl)-6-[3-(trifluoro­meth­yl)anilino]­pyrimidine-5-carbonitrile

**DOI:** 10.1107/S1600536812025895

**Published:** 2012-06-13

**Authors:** Ali A. El-Emam, Omar A. Al-Deeb, Nasser R. El-Brollosy, Seik Weng Ng, Edward R. T. Tiekink

**Affiliations:** a Department of Pharmaceutical Chemistry, College of Pharmacy, King Saud University, Riyadh 11451, Saudi Arabia; b Department of Chemistry, University of Malaya, 50603 Kuala Lumpur, Malaysia; c Chemistry Department, Faculty of Science, King Abdulaziz University, PO Box 80203 Jeddah, Saudi Arabia

## Abstract

Three independent mol­ecules comprise the asymmetric unit of the title compound, C_23_H_20_ClF_3_N_4_S. The conformations of the mol­ecules are similar with the chloro­benzene and CF_3_-benzene rings almost perpendicular to, and almost co-planar with, the pyrimidinyl ring [range of dihedral angles = 80.36 (13)–88.07 (14) and 11.89 (14)–23.30 (14)°, respectively]; the benzene rings are roughly orthogonal to each other [64.81 (16)–72.16 (15)°]. In the crystal, two of the independent mol­ecules associate *via* weak N—H⋯N(cyano) hydrogen bonds and 12-membered {⋯HNC_3_N}_2_ synthons; the third independent mol­ecule self-associates similarly but about a centre of inversion. The sample studied was found to be a non-merohedral twin and the minor twin component refined to 47.16 (7)%.

## Related literature

For the chemotherapeutic efficacy of pyrimidine derivatives, see: Al-Safarjalani *et al.* (2005 ▶); Brunelle *et al.* (2007 ▶); Ding *et al.* (2006 ▶); Al-Abdullah *et al.* (2011 ▶). For recent inter­est in the chemical and pharmacological properties of pyrimidine derivatives, see: Al-Omar *et al.* (2010 ▶); El-Emam *et al.* (2011 ▶). For the treatment of data from a twinned crystal, see: Spek (2009 ▶).
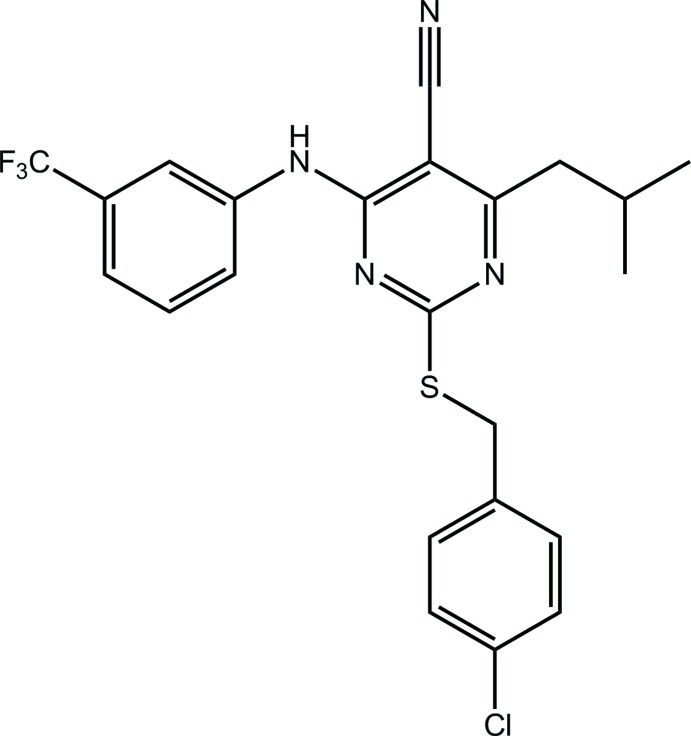


## Experimental

### 

#### Crystal data

C_23_H_20_ClF_3_N_4_S
*M* *_r_* = 476.94
Triclinic, 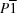

*a* = 15.3906 (6) Å
*b* = 15.7040 (7) Å
*c* = 15.9129 (7) Å
α = 89.568 (3)°
β = 74.825 (4)°
γ = 62.980 (4)°
*V* = 3278.9 (2) Å^3^
*Z* = 6
Cu *K*α radiation
μ = 2.83 mm^−1^
*T* = 100 K
0.35 × 0.15 × 0.03 mm

#### Data collection

Agilent SuperNova Dual diffractometer with Atlas detector
Absorption correction: multi-scan (*CrysAlis PRO*; Agilent, 2011 ▶) *T*_min_ = 0.437, *T*_max_ = 0.920
38203 measured reflections
25266 independent reflections
18393 reflections with *I* > 2σ(*I*)
*R*_int_ = 0.075

#### Refinement

*R*[*F* ^2^ > 2σ(*F* ^2^)] = 0.064
*wR*(*F* ^2^) = 0.190
*S* = 1.02
25266 reflections
866 parameters
H-atom parameters constrained
Δρ_max_ = 1.44 e Å^−3^
Δρ_min_ = −0.53 e Å^−3^

### 

Data collection: *CrysAlis PRO* (Agilent, 2011 ▶); cell refinement: *CrysAlis PRO*; data reduction: *CrysAlis PRO*; program(s) used to solve structure: *SHELXS97* (Sheldrick, 2008 ▶); program(s) used to refine structure: *SHELXL97* (Sheldrick, 2008 ▶); molecular graphics: *ORTEP-3* (Farrugia, 1997 ▶), *DIAMOND* (Brandenburg, 2006 ▶) and *QMol* (Gans & Shalloway, 2001 ▶); software used to prepare material for publication: *publCIF* (Westrip, 2010 ▶).

## Supplementary Material

Crystal structure: contains datablock(s) general, I. DOI: 10.1107/S1600536812025895/hb6786sup1.cif

Structure factors: contains datablock(s) I. DOI: 10.1107/S1600536812025895/hb6786Isup2.hkl

Supplementary material file. DOI: 10.1107/S1600536812025895/hb6786Isup3.cml

Additional supplementary materials:  crystallographic information; 3D view; checkCIF report

## Figures and Tables

**Table 1:** Hydrogen-bond geometry (Å, °)

*D*—H⋯*A*	*D*—H	H⋯*A*	*D*⋯*A*	*D*—H⋯*A*
N4—H4*n*⋯N7^i^	0.88	2.39	3.227 (3)	159
N8—H8*n*⋯N3^i^	0.88	2.38	3.213 (3)	158
N12—H12*n*⋯N11^ii^	0.88	2.36	3.206 (3)	160

Symmetry codes: (i) 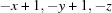; (ii) 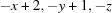.

**Fig. 1.**
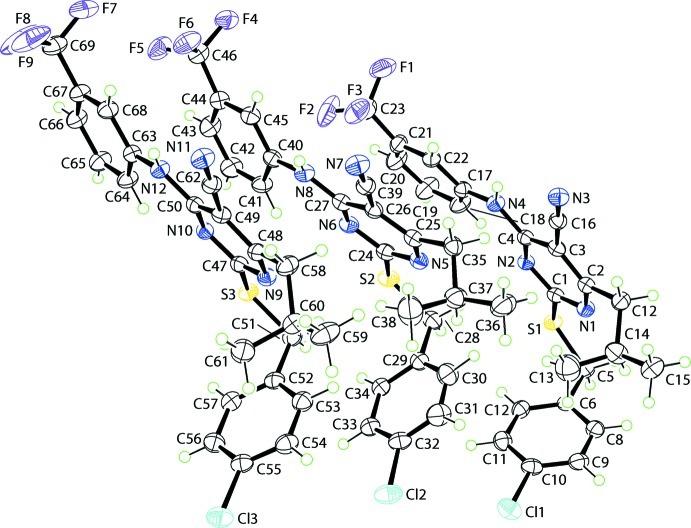
The molecular structures of the three independent molecules comprising (I) showing displacement ellipsoids at the 50% probability level.

**Fig. 2.**
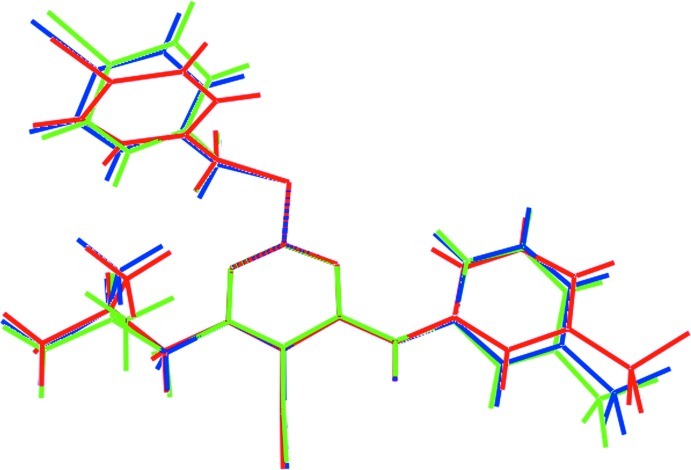
Overlay diagram of the three independent molecules comprising the asymmetric unit of (I). The first independent molecule (with the N1 atom) is shown in red, the second (N5) in blue and the third (N9) in green. The N—S—N atoms in each molecule have been overlapped.

**Fig. 3.**
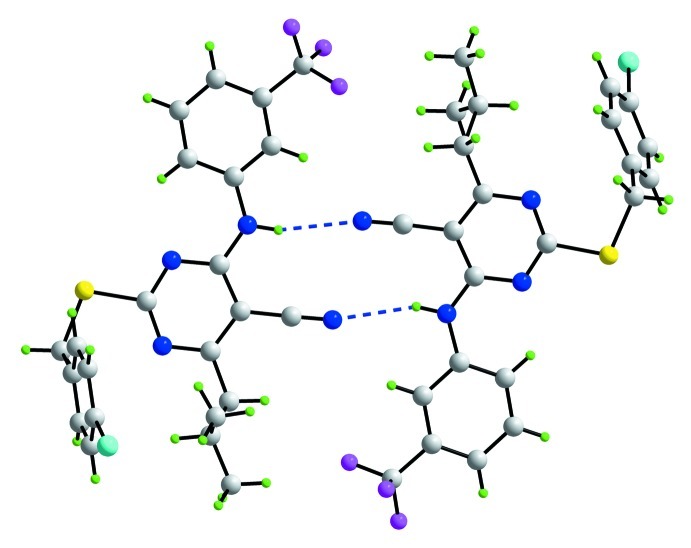
A view of a supramolecular dimeric aggregate in (I) where the first and second independent molecules are connected via N—H···N hydrogen bonds (dashed lines).

## supplementary crystallographic information

### Comment

The chemotherapeutic efficacy of pyrimidine derivatives is related to their ability to inhibit vital enzymes responsible for DNA bio-synthesis. Thus, several pyrimidine non-nucleoside derivatives are known to exhibit anti-cancer (Al-Safarjalani *et al.*, 2005), anti-viral (Brunelle *et al.*, 2007; Ding *et al.*, 2006) and anti-bacterial activities (Al-Abdullah *et al.*, 2011). In continuation to our interest in the chemical and pharmacological properties of pyrimidine derivatives (Al-Omar *et al.*, 2010; El-Emam *et al.* 2011), we synthesized the title compound (I) as potential a chemotherapeutic agent.

In (I), Fig. 1, three independent molecules comprise the asymmetric unit, and these have very similar molecular conformations as seen in the overlay diagram, Fig. 2. With respect to the pyrimidinyl ring, the chlorobenzene and CF_3_-benzene rings form dihedral angles of 80.36 (13) and 11.89 (14)°, respectively, for the N1-containing molecule; the dihedral angle between the benzene rings is 69.50 (14)°. For the N5-containing molecule the dihedral angles are 88.07 (14), 23.30 (14) and 64.81 (16), respectively, and for the N9-containing molecule, the angles are 85.94 (14), 13.81 (14) and 72.16 (15)°, respectively. In each case, the 2-methylpropyl group is orientated in the same direction as the chlorobenzene ring.

In the crystal structure, the N1- and N5-containing molecules associate to form a two molecule aggregate *via N*—*H*···*N*(cyano) hydrogen bonds and 12-membered {···HNC_3_N}_2_ synthons, Table 1 and Fig. 3. The N3-containing molecules self-associate similarly but over a centre of inversion, Table 1.

### Experimental

To a solution of 2-(4-chlorobenzylsulfanyl)-6-chloro-4-(2-methylpropyl)pyrimidine-5-carbonitrile (705 mg, 2 mmol) in ethanol (8 ml), 3-trifluoromethylaniline (0.002 mol) and anhydrous potassium carbonate (322 mg, 2 mmol) were added and the mixture was heated under reflux for 6 h. On cooling the mixture, the solvent was then distilled *in vacuo*, and water (10 ml) was added to the residue. The separated precipitate was filtered, washed with cold water, dried and crystallized from ethanol to yield 591 mg (62%) of the title compound as colourless crystals. *M*.pt: 409–411 K. Colourless prisms were obtained by slow evaporation of its CHCl_3_:EtOH (1:1, 5 ml) solution held at room temperature. ^1^H NMR (DMSO-d_6_, 500.13 MHz): δ 0.92 (d, 6H, CH_3_, J = 7.0 Hz), 2.08–2.12 (m, 1H, CH), 2.62 (d, 2H, CH_2_CH, J = 7.0 Hz), 4.24 (s, 2H, CH2S), 7.10–7.25 (m, 4H, Ar—H), 7.45–7.57 (m, 2H, Ar—H), 7.83–7.85 (m, 1H, Ar—H), 8.0 (s, 1H, Ar—H), 10.01 (s, 1H, NH). ^13^C NMR (DMSO-d_6_, 125.76 MHz): δ 21.88 (CH_3_CH), 27.68 (CHCH_3_), 33.06 (CH_2_S), 44.92 (CH_2_CH), 87.66 (C-5), 115.02 (CN), 115.65, 121.23, 124.08, 127.23, 128.65, 129.60, 129.75, 129.90, 131.25, 131.86, 137.23, 139.26 (Ar—C & CF_3_), 158.90 (C-6), 172.88, 173.28 (C-2 & C-4).

### Refinement

Carbon-bound H-atoms were placed in calculated positions [N—H = 0.88 Å and C—H = 0.95 to 1.00 Å, *U*_iso_(H) = 1.2–1.5*U*_eq_(N, C)] and were included in the refinement in the riding model approximation. The sample studied iss a non-merohedral twin and a full sphere of reflections was measured. As it was not possible to separate the diffraction spots in two domains, the twin domains were separated using the *TwinRotMat* routine of *PLATON* (Spek, 2009). The minor twin component refined to 47.16 (7)%. Two reflections,*i.e*. (11 8 2) and (0 2 4), were omitted owing to poor agreement. The maximum and minimum residual electron density peaks of 1.44 and 0.53 e Å^-3^, respectively, were located 0.51 Å and 0.81 Å from the H13C and F9 atoms, respectively.

### Crystal data

**Table:**

C_23_H_20_ClF_3_N_4_S	*Z* = 6
*M**_r_* = 476.94	*F*(000) = 1476
Triclinic, *P*1	*D*_x_ = 1.449 Mg m^−^^3^
Hall symbol: -P 1	Cu *K*α radiation, λ = 1.54184 Å
*a* = 15.3906 (6) Å	Cell parameters from 6525 reflections
*b* = 15.7040 (7) Å	θ = 2.9–76.4°
*c* = 15.9129 (7) Å	µ = 2.83 mm^−^^1^
α = 89.568 (3)°	*T* = 100 K
β = 74.825 (4)°	Prism, colourless
γ = 62.980 (4)°	0.35 × 0.15 × 0.03 mm
*V* = 3278.9 (2) Å^3^	

### Data collection

**Table:**

Agilent SuperNova Dual diffractometer with Atlas detector	25266 independent reflections
Radiation source: SuperNova (Cu) X-ray Source	18393 reflections with *I* > 2σ(*I*)
Mirror monochromator	*R*_int_ = 0.075
Detector resolution: 10.4041 pixels mm^-1^	θ_max_ = 77.2°, θ_min_ = 2.9°
ω scan	*h* = −19→14
Absorption correction: multi-scan (*CrysAlis PRO*; Agilent, 2011)	*k* = −19→19
*T*_min_ = 0.437, *T*_max_ = 0.920	*l* = −20→20
38203 measured reflections	

### Refinement

**Table:**

Refinement on *F*^2^	Primary atom site location: structure-invariant direct methods
Least-squares matrix: full	Secondary atom site location: difference Fourier map
*R*[*F*^2^ > 2σ(*F*^2^)] = 0.064	Hydrogen site location: inferred from neighbouring sites
*wR*(*F*^2^) = 0.190	H-atom parameters constrained
*S* = 1.02	*w* = 1/[σ^2^(*F*_o_^2^) + (0.1074*P*)^2^ + 1.9445*P*] where *P* = (*F*_o_^2^ + 2*F*_c_^2^)/3
25266 reflections	(Δ/σ)_max_ = 0.001
866 parameters	Δρ_max_ = 1.44 e Å^−^^3^
0 restraints	Δρ_min_ = −0.53 e Å^−^^3^

### Special details

**Table:**

Geometry. All e.s.d.'s (except the e.s.d. in the dihedral angle between two l.s. planes) are estimated using the full covariance matrix. The cell e.s.d.'s are taken into account individually in the estimation of e.s.d.'s in distances, angles and torsion angles; correlations between e.s.d.'s in cell parameters are only used when they are defined by crystal symmetry. An approximate (isotropic) treatment of cell e.s.d.'s is used for estimating e.s.d.'s involving l.s. planes.
Refinement. Refinement of *F*^2^ against ALL reflections. The weighted *R*-factor *wR* and goodness of fit *S* are based on *F*^2^, conventional *R*-factors *R* are based on *F*, with *F* set to zero for negative *F*^2^. The threshold expression of *F*^2^ > σ(*F*^2^) is used only for calculating *R*-factors(gt) *etc*. and is not relevant to the choice of reflections for refinement. *R*-factors based on *F*^2^ are statistically about twice as large as those based on *F*, and *R*- factors based on ALL data will be even larger.

### Fractional atomic coordinates and isotropic or equivalent isotropic displacement parameters (Å^2^)

**Table:**

	*x*	*y*	*z*	*U*_iso_*/*U*_eq_	
Cl1	0.28241 (5)	0.07400 (5)	0.46993 (5)	0.04104 (17)	
Cl2	0.54081 (6)	0.09270 (5)	0.45600 (5)	0.04320 (17)	
Cl3	0.88514 (6)	0.05812 (5)	0.48589 (5)	0.04180 (17)	
S1	−0.09695 (4)	0.50003 (4)	0.38610 (4)	0.02693 (13)	
S2	0.26108 (5)	0.55904 (5)	0.35580 (4)	0.03043 (14)	
S3	0.59118 (4)	0.50668 (4)	0.36852 (4)	0.02694 (13)	
F1	0.17005 (17)	0.91092 (16)	0.12971 (16)	0.0647 (6)	
F2	0.2082 (2)	0.9126 (2)	0.24916 (15)	0.0777 (8)	
F3	0.30080 (15)	0.79316 (15)	0.14940 (14)	0.0566 (5)	
F4	0.48491 (15)	0.96664 (14)	0.10659 (13)	0.0503 (5)	
F5	0.55114 (16)	0.98424 (14)	0.20327 (12)	0.0502 (5)	
F6	0.63952 (13)	0.86860 (13)	0.09720 (12)	0.0467 (4)	
F7	0.7953 (2)	0.9366 (2)	0.10386 (19)	0.0935 (10)	
F8	0.8286 (3)	0.9722 (2)	0.21371 (15)	0.1110 (13)	
F9	0.93890 (18)	0.85381 (17)	0.12129 (18)	0.0814 (8)	
N1	0.03473 (16)	0.38490 (15)	0.23888 (13)	0.0263 (4)	
N2	0.02592 (15)	0.53909 (14)	0.26904 (13)	0.0237 (4)	
N3	0.27750 (16)	0.40345 (16)	−0.01072 (14)	0.0296 (5)	
N4	0.12993 (15)	0.58965 (15)	0.17176 (13)	0.0258 (4)	
H4n	0.1760	0.5755	0.1205	0.031*	
N5	0.38696 (16)	0.44653 (16)	0.20522 (13)	0.0274 (4)	
N6	0.40376 (16)	0.58285 (15)	0.25038 (13)	0.0273 (4)	
N7	0.67800 (18)	0.43372 (16)	−0.01233 (15)	0.0344 (5)	
N8	0.53033 (16)	0.61660 (15)	0.16659 (13)	0.0272 (4)	
H8n	0.5864	0.5944	0.1224	0.033*	
N9	0.73047 (16)	0.39133 (15)	0.22323 (13)	0.0261 (4)	
N10	0.72270 (15)	0.54339 (14)	0.26119 (13)	0.0229 (4)	
N11	0.98810 (17)	0.41309 (16)	−0.00944 (14)	0.0316 (5)	
N12	0.83306 (15)	0.59339 (14)	0.17164 (13)	0.0242 (4)	
H12n	0.8852	0.5766	0.1243	0.029*	
C1	−0.00049 (17)	0.46909 (17)	0.28614 (15)	0.0242 (5)	
C2	0.10854 (18)	0.36623 (18)	0.16294 (16)	0.0256 (5)	
C3	0.14142 (18)	0.43437 (18)	0.13701 (15)	0.0249 (5)	
C4	0.09820 (18)	0.52258 (17)	0.19325 (15)	0.0236 (5)	
C5	−0.09619 (19)	0.38575 (18)	0.40321 (16)	0.0272 (5)	
H5A	−0.1556	0.3967	0.4534	0.033*	
H5B	−0.1047	0.3610	0.3505	0.033*	
C6	−0.00106 (19)	0.30952 (18)	0.42101 (15)	0.0266 (5)	
C7	0.0331 (2)	0.21336 (19)	0.39186 (17)	0.0321 (5)	
H7	−0.0040	0.1972	0.3614	0.038*	
C8	0.1204 (2)	0.14040 (19)	0.40635 (17)	0.0328 (6)	
H8A	0.1431	0.0749	0.3862	0.039*	
C9	0.1735 (2)	0.16499 (19)	0.45067 (17)	0.0310 (5)	
C10	0.1415 (2)	0.2598 (2)	0.48143 (17)	0.0325 (6)	
H10	0.1785	0.2755	0.5124	0.039*	
C11	0.0541 (2)	0.33159 (18)	0.46595 (17)	0.0292 (5)	
H11	0.0315	0.3970	0.4864	0.035*	
C12	0.1518 (2)	0.27018 (18)	0.10984 (16)	0.0292 (5)	
H12A	0.2077	0.2639	0.0581	0.035*	
H12B	0.0981	0.2680	0.0881	0.035*	
C13	0.2646 (3)	0.1856 (3)	0.2067 (3)	0.0562 (9)	
H13A	0.2868	0.1291	0.2381	0.084*	
H13B	0.2302	0.2444	0.2488	0.084*	
H13C	0.3241	0.1845	0.1639	0.084*	
C14	0.1932 (2)	0.1840 (2)	0.1604 (2)	0.0395 (6)	
H14	0.1329	0.1882	0.2070	0.047*	
C15	0.2373 (2)	0.0905 (2)	0.1034 (2)	0.0397 (6)	
H15A	0.2627	0.0371	0.1377	0.060*	
H15B	0.2936	0.0852	0.0537	0.060*	
H15C	0.1845	0.0880	0.0815	0.060*	
C16	0.21728 (19)	0.41608 (17)	0.05535 (16)	0.0261 (5)	
C17	0.10026 (19)	0.67876 (18)	0.21925 (16)	0.0266 (5)	
C18	0.0161 (2)	0.7249 (2)	0.29302 (18)	0.0363 (6)	
H18	−0.0274	0.6970	0.3149	0.044*	
C19	−0.0034 (3)	0.8118 (2)	0.3341 (2)	0.0450 (8)	
H19	−0.0602	0.8424	0.3848	0.054*	
C20	0.0572 (2)	0.8555 (2)	0.30360 (19)	0.0421 (7)	
H20	0.0426	0.9151	0.3327	0.051*	
C21	0.1402 (2)	0.8094 (2)	0.22893 (17)	0.0327 (6)	
C22	0.16137 (19)	0.72244 (19)	0.18721 (16)	0.0281 (5)	
H22	0.2180	0.6921	0.1363	0.034*	
C23	0.2036 (2)	0.8567 (2)	0.1902 (2)	0.0416 (7)	
C24	0.36230 (18)	0.52357 (18)	0.25885 (16)	0.0270 (5)	
C25	0.46783 (18)	0.42328 (18)	0.13324 (16)	0.0265 (5)	
C26	0.52042 (18)	0.47668 (18)	0.12004 (16)	0.0251 (5)	
C27	0.48436 (18)	0.56034 (18)	0.17990 (16)	0.0261 (5)	
C28	0.23542 (19)	0.4576 (2)	0.36258 (17)	0.0317 (6)	
H28A	0.1690	0.4771	0.4071	0.038*	
H28B	0.2290	0.4413	0.3053	0.038*	
C29	0.31508 (19)	0.36827 (19)	0.38604 (16)	0.0282 (5)	
C30	0.3587 (2)	0.2816 (2)	0.33236 (19)	0.0410 (7)	
H30	0.3405	0.2805	0.2799	0.049*	
C31	0.4279 (3)	0.1970 (2)	0.35333 (19)	0.0432 (7)	
H31	0.4561	0.1379	0.3164	0.052*	
C32	0.4553 (2)	0.19952 (19)	0.42846 (17)	0.0322 (6)	
C33	0.41735 (19)	0.28512 (19)	0.48200 (17)	0.0289 (5)	
H33	0.4393	0.2864	0.5323	0.035*	
C34	0.34619 (18)	0.36938 (18)	0.46041 (16)	0.0267 (5)	
H34	0.3185	0.4285	0.4971	0.032*	
C35	0.4953 (2)	0.33835 (19)	0.07026 (17)	0.0298 (5)	
H35A	0.5464	0.3356	0.0161	0.036*	
H35B	0.4337	0.3482	0.0541	0.036*	
C36	0.5468 (3)	0.1638 (2)	0.0437 (2)	0.0517 (9)	
H36A	0.5728	0.1024	0.0679	0.078*	
H36B	0.5939	0.1578	−0.0136	0.078*	
H36C	0.4797	0.1804	0.0369	0.078*	
C37	0.5375 (2)	0.2426 (2)	0.10586 (19)	0.0399 (7)	
H37	0.4883	0.2483	0.1635	0.048*	
C38	0.6384 (3)	0.2182 (3)	0.1213 (3)	0.0643 (11)	
H38A	0.6297	0.2696	0.1623	0.096*	
H38B	0.6880	0.2120	0.0654	0.096*	
H38C	0.6630	0.1572	0.1461	0.096*	
C39	0.60785 (19)	0.45106 (18)	0.04593 (17)	0.0280 (5)	
C40	0.50169 (19)	0.70637 (18)	0.21335 (16)	0.0271 (5)	
C41	0.4449 (2)	0.73593 (19)	0.30145 (17)	0.0312 (5)	
H41	0.4219	0.6954	0.3341	0.037*	
C42	0.4221 (2)	0.8246 (2)	0.34108 (18)	0.0377 (6)	
H42	0.3828	0.8448	0.4009	0.045*	
C43	0.4557 (2)	0.8845 (2)	0.29504 (18)	0.0359 (6)	
H43	0.4398	0.9453	0.3227	0.043*	
C44	0.51292 (19)	0.85383 (18)	0.20761 (17)	0.0293 (5)	
C45	0.53599 (19)	0.76585 (18)	0.16691 (16)	0.0280 (5)	
H45	0.5754	0.7459	0.1071	0.034*	
C46	0.5468 (2)	0.9177 (2)	0.15434 (18)	0.0341 (6)	
C47	0.69373 (18)	0.47470 (17)	0.27320 (16)	0.0244 (5)	
C48	0.80934 (19)	0.37318 (18)	0.15126 (16)	0.0253 (5)	
C49	0.84461 (18)	0.44015 (17)	0.13139 (15)	0.0244 (5)	
C50	0.79899 (17)	0.52733 (17)	0.18920 (15)	0.0225 (5)	
C51	0.57135 (19)	0.40147 (19)	0.37399 (18)	0.0301 (5)	
H51A	0.5033	0.4196	0.4152	0.036*	
H51B	0.5708	0.3818	0.3153	0.036*	
C52	0.64989 (18)	0.31549 (18)	0.40284 (16)	0.0272 (5)	
C53	0.6869 (2)	0.2237 (2)	0.36093 (18)	0.0326 (6)	
H53	0.6631	0.2152	0.3138	0.039*	
C54	0.7585 (2)	0.14369 (19)	0.38691 (18)	0.0343 (6)	
H54	0.7831	0.0808	0.3584	0.041*	
C55	0.7929 (2)	0.15728 (19)	0.45457 (17)	0.0302 (5)	
C56	0.7568 (2)	0.2475 (2)	0.49877 (17)	0.0322 (5)	
H56	0.7804	0.2554	0.5461	0.039*	
C57	0.68469 (19)	0.32682 (19)	0.47215 (17)	0.0295 (5)	
H57	0.6590	0.3895	0.5018	0.035*	
C58	0.8553 (2)	0.27941 (18)	0.09367 (17)	0.0322 (6)	
H58A	0.9213	0.2686	0.0529	0.039*	
H58B	0.8098	0.2845	0.0578	0.039*	
C59	0.9241 (3)	0.1005 (2)	0.0764 (2)	0.0438 (7)	
H59A	0.8819	0.1086	0.0374	0.066*	
H59B	0.9312	0.0446	0.1074	0.066*	
H59C	0.9916	0.0904	0.0417	0.066*	
C60	0.8735 (2)	0.19117 (19)	0.14328 (17)	0.0307 (5)	
H60	0.8057	0.1991	0.1790	0.037*	
C61	0.9363 (2)	0.1818 (2)	0.2048 (2)	0.0437 (7)	
H61A	0.9021	0.2405	0.2469	0.066*	
H61B	1.0037	0.1726	0.1709	0.066*	
H61C	0.9438	0.1263	0.2364	0.066*	
C62	0.92467 (19)	0.42335 (17)	0.05287 (16)	0.0257 (5)	
C63	0.79769 (18)	0.68498 (17)	0.21762 (16)	0.0242 (5)	
C64	0.7342 (2)	0.71623 (19)	0.30330 (16)	0.0303 (5)	
H64	0.7108	0.6754	0.3347	0.036*	
C65	0.7051 (2)	0.8073 (2)	0.34271 (18)	0.0344 (6)	
H65	0.6613	0.8282	0.4012	0.041*	
C66	0.7384 (2)	0.86838 (19)	0.29861 (17)	0.0315 (5)	
H66	0.7174	0.9309	0.3260	0.038*	
C67	0.8028 (2)	0.83617 (19)	0.21389 (17)	0.0297 (5)	
C68	0.83219 (19)	0.74554 (18)	0.17302 (16)	0.0283 (5)	
H68	0.8759	0.7248	0.1145	0.034*	
C69	0.8404 (2)	0.8999 (2)	0.16351 (18)	0.0380 (6)	

### Atomic displacement parameters (Å^2^)

**Table:**

	*U*^11^	*U*^22^	*U*^33^	*U*^12^	*U*^13^	*U*^23^
Cl1	0.0405 (4)	0.0330 (3)	0.0356 (3)	−0.0047 (3)	−0.0127 (3)	0.0000 (3)
Cl2	0.0407 (4)	0.0286 (3)	0.0438 (4)	−0.0078 (3)	−0.0023 (3)	0.0072 (3)
Cl3	0.0428 (4)	0.0302 (3)	0.0434 (4)	−0.0096 (3)	−0.0128 (3)	0.0082 (3)
S1	0.0247 (3)	0.0270 (3)	0.0246 (3)	−0.0116 (2)	−0.0014 (2)	0.0035 (2)
S2	0.0274 (3)	0.0317 (3)	0.0238 (3)	−0.0099 (2)	−0.0017 (2)	0.0056 (2)
S3	0.0243 (3)	0.0263 (3)	0.0262 (3)	−0.0116 (2)	−0.0016 (2)	0.0036 (2)
F1	0.0703 (14)	0.0676 (14)	0.0796 (15)	−0.0485 (12)	−0.0286 (12)	0.0407 (12)
F2	0.1067 (19)	0.1026 (19)	0.0552 (13)	−0.0886 (17)	0.0028 (12)	−0.0138 (12)
F3	0.0438 (10)	0.0621 (13)	0.0698 (13)	−0.0359 (10)	−0.0047 (9)	0.0128 (10)
F4	0.0558 (11)	0.0463 (10)	0.0636 (12)	−0.0297 (9)	−0.0300 (10)	0.0288 (9)
F5	0.0718 (13)	0.0456 (10)	0.0407 (10)	−0.0399 (10)	−0.0046 (9)	−0.0026 (8)
F6	0.0421 (10)	0.0383 (9)	0.0470 (10)	−0.0198 (8)	0.0086 (8)	0.0023 (8)
F7	0.121 (2)	0.124 (2)	0.108 (2)	−0.098 (2)	−0.0713 (19)	0.0905 (19)
F8	0.214 (4)	0.096 (2)	0.0456 (13)	−0.126 (2)	0.0278 (17)	−0.0165 (12)
F9	0.0590 (14)	0.0630 (14)	0.104 (2)	−0.0338 (12)	0.0138 (13)	0.0301 (13)
N1	0.0272 (10)	0.0292 (10)	0.0217 (10)	−0.0142 (9)	−0.0037 (8)	0.0032 (8)
N2	0.0210 (9)	0.0258 (10)	0.0222 (10)	−0.0103 (8)	−0.0044 (8)	0.0039 (8)
N3	0.0290 (11)	0.0293 (11)	0.0254 (11)	−0.0117 (9)	−0.0036 (9)	0.0027 (8)
N4	0.0245 (10)	0.0273 (10)	0.0223 (10)	−0.0123 (8)	−0.0012 (8)	0.0018 (8)
N5	0.0257 (10)	0.0318 (11)	0.0238 (10)	−0.0127 (9)	−0.0074 (8)	0.0057 (8)
N6	0.0259 (10)	0.0278 (10)	0.0228 (10)	−0.0096 (8)	−0.0043 (8)	0.0036 (8)
N7	0.0350 (12)	0.0298 (11)	0.0294 (12)	−0.0128 (10)	0.0001 (10)	0.0012 (9)
N8	0.0257 (10)	0.0262 (10)	0.0233 (10)	−0.0099 (8)	−0.0012 (8)	0.0013 (8)
N9	0.0269 (10)	0.0259 (10)	0.0250 (10)	−0.0124 (8)	−0.0065 (8)	0.0034 (8)
N10	0.0219 (9)	0.0247 (10)	0.0217 (10)	−0.0108 (8)	−0.0056 (8)	0.0034 (8)
N11	0.0341 (12)	0.0277 (11)	0.0278 (11)	−0.0140 (9)	−0.0016 (9)	0.0009 (8)
N12	0.0252 (10)	0.0232 (10)	0.0209 (9)	−0.0115 (8)	−0.0013 (8)	0.0003 (7)
C1	0.0217 (11)	0.0268 (12)	0.0222 (11)	−0.0093 (9)	−0.0071 (9)	0.0064 (9)
C2	0.0266 (12)	0.0269 (12)	0.0238 (12)	−0.0118 (10)	−0.0095 (10)	0.0056 (9)
C3	0.0238 (11)	0.0277 (12)	0.0210 (11)	−0.0102 (10)	−0.0064 (9)	0.0041 (9)
C4	0.0213 (11)	0.0269 (12)	0.0228 (11)	−0.0111 (9)	−0.0070 (9)	0.0061 (9)
C5	0.0271 (12)	0.0275 (12)	0.0271 (12)	−0.0161 (10)	−0.0016 (10)	0.0046 (9)
C6	0.0295 (12)	0.0287 (12)	0.0203 (11)	−0.0150 (10)	−0.0027 (9)	0.0060 (9)
C7	0.0408 (14)	0.0320 (13)	0.0271 (13)	−0.0196 (12)	−0.0108 (11)	0.0057 (10)
C8	0.0449 (15)	0.0256 (12)	0.0259 (13)	−0.0158 (12)	−0.0085 (11)	0.0039 (10)
C9	0.0337 (13)	0.0284 (13)	0.0230 (12)	−0.0103 (11)	−0.0040 (10)	0.0047 (10)
C10	0.0328 (13)	0.0348 (14)	0.0291 (13)	−0.0157 (11)	−0.0079 (11)	0.0019 (10)
C11	0.0316 (13)	0.0262 (12)	0.0280 (13)	−0.0141 (10)	−0.0047 (10)	0.0004 (10)
C12	0.0330 (13)	0.0295 (13)	0.0247 (12)	−0.0164 (11)	−0.0043 (10)	0.0014 (10)
C13	0.052 (2)	0.053 (2)	0.061 (2)	−0.0204 (17)	−0.0201 (17)	0.0047 (17)
C14	0.0391 (15)	0.0330 (14)	0.0354 (15)	−0.0076 (12)	−0.0114 (12)	0.0006 (12)
C15	0.0395 (15)	0.0310 (14)	0.0473 (17)	−0.0174 (12)	−0.0091 (13)	0.0054 (12)
C16	0.0276 (12)	0.0242 (11)	0.0254 (12)	−0.0111 (10)	−0.0080 (10)	0.0033 (9)
C17	0.0278 (12)	0.0278 (12)	0.0242 (12)	−0.0134 (10)	−0.0067 (10)	0.0045 (9)
C18	0.0393 (15)	0.0348 (14)	0.0317 (14)	−0.0214 (12)	0.0021 (12)	0.0010 (11)
C19	0.0502 (18)	0.0415 (16)	0.0342 (15)	−0.0260 (15)	0.0109 (13)	−0.0085 (12)
C20	0.0521 (18)	0.0383 (16)	0.0337 (15)	−0.0265 (14)	0.0017 (13)	−0.0074 (12)
C21	0.0390 (14)	0.0353 (14)	0.0299 (13)	−0.0237 (12)	−0.0078 (11)	0.0037 (11)
C22	0.0289 (12)	0.0332 (13)	0.0228 (12)	−0.0170 (11)	−0.0041 (10)	0.0042 (10)
C23	0.0485 (17)	0.0475 (17)	0.0375 (15)	−0.0325 (15)	−0.0073 (13)	0.0030 (13)
C24	0.0219 (11)	0.0311 (12)	0.0242 (12)	−0.0096 (10)	−0.0061 (9)	0.0071 (10)
C25	0.0252 (12)	0.0295 (12)	0.0222 (11)	−0.0101 (10)	−0.0080 (9)	0.0055 (9)
C26	0.0231 (11)	0.0285 (12)	0.0222 (11)	−0.0110 (10)	−0.0062 (9)	0.0042 (9)
C27	0.0244 (11)	0.0274 (12)	0.0223 (11)	−0.0085 (10)	−0.0071 (9)	0.0044 (9)
C28	0.0256 (12)	0.0444 (15)	0.0261 (13)	−0.0177 (11)	−0.0066 (10)	0.0093 (11)
C29	0.0244 (12)	0.0364 (14)	0.0242 (12)	−0.0166 (11)	−0.0035 (9)	0.0065 (10)
C30	0.0520 (18)	0.0446 (17)	0.0280 (14)	−0.0248 (15)	−0.0095 (13)	0.0003 (12)
C31	0.0567 (19)	0.0321 (15)	0.0314 (15)	−0.0171 (14)	−0.0049 (13)	−0.0048 (11)
C32	0.0303 (13)	0.0297 (13)	0.0289 (13)	−0.0122 (11)	0.0001 (10)	0.0055 (10)
C33	0.0270 (12)	0.0315 (13)	0.0255 (12)	−0.0144 (10)	−0.0022 (10)	0.0038 (10)
C34	0.0229 (11)	0.0279 (12)	0.0263 (12)	−0.0120 (10)	−0.0019 (9)	0.0018 (9)
C35	0.0291 (12)	0.0367 (14)	0.0249 (12)	−0.0168 (11)	−0.0073 (10)	0.0035 (10)
C36	0.083 (3)	0.0443 (18)	0.0380 (17)	−0.0402 (18)	−0.0142 (16)	0.0065 (14)
C37	0.0517 (18)	0.0362 (15)	0.0330 (15)	−0.0217 (14)	−0.0122 (13)	0.0052 (12)
C38	0.066 (2)	0.0346 (17)	0.095 (3)	−0.0128 (17)	−0.048 (2)	0.0052 (18)
C39	0.0310 (13)	0.0238 (12)	0.0280 (13)	−0.0113 (10)	−0.0094 (11)	0.0034 (9)
C40	0.0258 (12)	0.0279 (12)	0.0232 (12)	−0.0092 (10)	−0.0065 (9)	0.0027 (9)
C41	0.0360 (14)	0.0310 (13)	0.0233 (12)	−0.0155 (11)	−0.0040 (10)	0.0048 (10)
C42	0.0429 (16)	0.0387 (15)	0.0245 (13)	−0.0185 (13)	0.0002 (11)	−0.0021 (11)
C43	0.0379 (15)	0.0293 (13)	0.0330 (14)	−0.0144 (12)	−0.0011 (11)	−0.0056 (11)
C44	0.0284 (12)	0.0270 (12)	0.0288 (13)	−0.0113 (10)	−0.0056 (10)	0.0026 (10)
C45	0.0246 (12)	0.0303 (13)	0.0238 (12)	−0.0106 (10)	−0.0029 (9)	0.0026 (9)
C46	0.0349 (14)	0.0308 (13)	0.0330 (14)	−0.0154 (11)	−0.0040 (11)	0.0002 (11)
C47	0.0231 (11)	0.0254 (11)	0.0244 (11)	−0.0103 (9)	−0.0085 (9)	0.0065 (9)
C48	0.0282 (12)	0.0257 (12)	0.0232 (11)	−0.0133 (10)	−0.0080 (10)	0.0054 (9)
C49	0.0254 (11)	0.0238 (11)	0.0218 (11)	−0.0101 (9)	−0.0060 (9)	0.0036 (9)
C50	0.0209 (11)	0.0240 (11)	0.0216 (11)	−0.0094 (9)	−0.0068 (9)	0.0043 (9)
C51	0.0231 (12)	0.0338 (13)	0.0336 (14)	−0.0149 (11)	−0.0056 (10)	0.0082 (11)
C52	0.0243 (12)	0.0290 (12)	0.0255 (12)	−0.0130 (10)	−0.0022 (9)	0.0067 (10)
C53	0.0355 (14)	0.0348 (14)	0.0282 (13)	−0.0186 (12)	−0.0063 (11)	0.0043 (11)
C54	0.0402 (15)	0.0268 (13)	0.0312 (14)	−0.0148 (11)	−0.0048 (11)	0.0014 (10)
C55	0.0274 (12)	0.0290 (13)	0.0287 (13)	−0.0119 (10)	−0.0021 (10)	0.0059 (10)
C56	0.0334 (13)	0.0344 (14)	0.0262 (13)	−0.0150 (11)	−0.0065 (10)	0.0040 (10)
C57	0.0295 (12)	0.0278 (12)	0.0276 (12)	−0.0135 (10)	−0.0022 (10)	0.0005 (10)
C58	0.0403 (14)	0.0265 (13)	0.0284 (13)	−0.0176 (11)	−0.0042 (11)	0.0007 (10)
C59	0.0546 (19)	0.0294 (14)	0.0396 (16)	−0.0208 (14)	0.0009 (14)	0.0007 (12)
C60	0.0303 (13)	0.0271 (13)	0.0332 (13)	−0.0142 (11)	−0.0055 (11)	0.0033 (10)
C61	0.0409 (16)	0.0335 (15)	0.059 (2)	−0.0153 (13)	−0.0221 (15)	0.0100 (13)
C62	0.0300 (12)	0.0197 (11)	0.0262 (12)	−0.0107 (9)	−0.0080 (10)	0.0022 (9)
C63	0.0244 (11)	0.0235 (11)	0.0235 (11)	−0.0110 (9)	−0.0058 (9)	0.0025 (9)
C64	0.0373 (14)	0.0287 (13)	0.0238 (12)	−0.0182 (11)	−0.0021 (10)	0.0023 (10)
C65	0.0387 (14)	0.0347 (14)	0.0255 (13)	−0.0199 (12)	0.0023 (11)	−0.0010 (10)
C66	0.0355 (14)	0.0277 (13)	0.0286 (13)	−0.0165 (11)	−0.0022 (11)	−0.0003 (10)
C67	0.0324 (13)	0.0281 (12)	0.0272 (13)	−0.0159 (11)	−0.0034 (10)	0.0047 (10)
C68	0.0302 (12)	0.0301 (13)	0.0211 (11)	−0.0144 (10)	−0.0011 (10)	0.0017 (9)
C69	0.0503 (17)	0.0345 (14)	0.0268 (13)	−0.0243 (13)	0.0008 (12)	0.0018 (11)

### Geometric parameters (Å, º)

**Table:**

Cl1—C9	1.741 (3)	C22—H22	0.9500
Cl2—C32	1.738 (3)	C25—C26	1.388 (3)
Cl3—C55	1.743 (3)	C25—C35	1.499 (4)
S1—C1	1.758 (2)	C26—C27	1.422 (3)
S1—C5	1.808 (2)	C26—C39	1.431 (3)
S2—C24	1.756 (2)	C28—C29	1.510 (4)
S2—C28	1.803 (3)	C28—H28A	0.9900
S3—C47	1.756 (2)	C28—H28B	0.9900
S3—C51	1.809 (3)	C29—C30	1.385 (4)
F1—C23	1.328 (4)	C29—C34	1.391 (4)
F2—C23	1.327 (4)	C30—C31	1.381 (4)
F3—C23	1.342 (4)	C30—H30	0.9500
F4—C46	1.334 (3)	C31—C32	1.375 (4)
F5—C46	1.343 (3)	C31—H31	0.9500
F6—C46	1.338 (3)	C32—C33	1.385 (4)
F7—C69	1.300 (4)	C33—C34	1.393 (4)
F8—C69	1.309 (4)	C33—H33	0.9500
F9—C69	1.323 (4)	C34—H34	0.9500
N1—C1	1.326 (3)	C35—C37	1.515 (4)
N1—C2	1.349 (3)	C35—H35A	0.9900
N2—C1	1.338 (3)	C35—H35B	0.9900
N2—C4	1.341 (3)	C36—C37	1.521 (4)
N3—C16	1.153 (3)	C36—H36A	0.9800
N4—C4	1.356 (3)	C36—H36B	0.9800
N4—C17	1.412 (3)	C36—H36C	0.9800
N4—H4n	0.8800	C37—C38	1.508 (5)
N5—C24	1.328 (3)	C37—H37	1.0000
N5—C25	1.357 (3)	C38—H38A	0.9800
N6—C24	1.336 (3)	C38—H38B	0.9800
N6—C27	1.342 (3)	C38—H38C	0.9800
N7—C39	1.145 (3)	C40—C45	1.390 (3)
N8—C27	1.346 (3)	C40—C41	1.395 (3)
N8—C40	1.419 (3)	C41—C42	1.383 (4)
N8—H8n	0.8800	C41—H41	0.9500
N9—C47	1.332 (3)	C42—C43	1.387 (4)
N9—C48	1.352 (3)	C42—H42	0.9500
N10—C50	1.336 (3)	C43—C44	1.388 (4)
N10—C47	1.337 (3)	C43—H43	0.9500
N11—C62	1.147 (3)	C44—C45	1.379 (4)
N12—C50	1.357 (3)	C44—C46	1.493 (4)
N12—C63	1.413 (3)	C45—H45	0.9500
N12—H12n	0.8800	C48—C49	1.387 (3)
C2—C3	1.396 (3)	C48—C58	1.498 (3)
C2—C12	1.498 (3)	C49—C50	1.422 (3)
C3—C4	1.426 (3)	C49—C62	1.433 (3)
C3—C16	1.429 (3)	C51—C52	1.514 (3)
C5—C6	1.509 (3)	C51—H51A	0.9900
C5—H5A	0.9900	C51—H51B	0.9900
C5—H5B	0.9900	C52—C53	1.385 (4)
C6—C7	1.391 (4)	C52—C57	1.391 (4)
C6—C11	1.393 (4)	C53—C54	1.391 (4)
C7—C8	1.389 (4)	C53—H53	0.9500
C7—H7	0.9500	C54—C55	1.375 (4)
C8—C9	1.381 (4)	C54—H54	0.9500
C8—H8A	0.9500	C55—C56	1.381 (4)
C9—C10	1.385 (4)	C56—C57	1.394 (4)
C10—C11	1.391 (4)	C56—H56	0.9500
C10—H10	0.9500	C57—H57	0.9500
C11—H11	0.9500	C58—C60	1.539 (4)
C12—C14	1.534 (4)	C58—H58A	0.9900
C12—H12A	0.9900	C58—H58B	0.9900
C12—H12B	0.9900	C59—C60	1.531 (4)
C13—C14	1.485 (5)	C59—H59A	0.9800
C13—H13A	0.9800	C59—H59B	0.9800
C13—H13B	0.9800	C59—H59C	0.9800
C13—H13C	0.9800	C60—C61	1.509 (4)
C14—C15	1.492 (4)	C60—H60	1.0000
C14—H14	1.0000	C61—H61A	0.9800
C15—H15A	0.9800	C61—H61B	0.9800
C15—H15B	0.9800	C61—H61C	0.9800
C15—H15C	0.9800	C63—C64	1.390 (3)
C17—C22	1.395 (3)	C63—C68	1.393 (3)
C17—C18	1.395 (4)	C64—C65	1.386 (4)
C18—C19	1.385 (4)	C64—H64	0.9500
C18—H18	0.9500	C65—C66	1.388 (4)
C19—C20	1.383 (4)	C65—H65	0.9500
C19—H19	0.9500	C66—C67	1.382 (4)
C20—C21	1.395 (4)	C66—H66	0.9500
C20—H20	0.9500	C67—C68	1.389 (4)
C21—C22	1.381 (4)	C67—C69	1.500 (3)
C21—C23	1.490 (4)	C68—H68	0.9500
			
C1—S1—C5	101.53 (11)	C29—C34—H34	119.5
C24—S2—C28	102.77 (13)	C33—C34—H34	119.5
C47—S3—C51	103.16 (12)	C25—C35—C37	114.1 (2)
C1—N1—C2	115.7 (2)	C25—C35—H35A	108.7
C1—N2—C4	116.0 (2)	C37—C35—H35A	108.7
C4—N4—C17	129.8 (2)	C25—C35—H35B	108.7
C4—N4—H4n	115.1	C37—C35—H35B	108.7
C17—N4—H4n	115.1	H35A—C35—H35B	107.6
C24—N5—C25	115.1 (2)	C37—C36—H36A	109.5
C24—N6—C27	116.4 (2)	C37—C36—H36B	109.5
C27—N8—C40	129.6 (2)	H36A—C36—H36B	109.5
C27—N8—H8n	115.2	C37—C36—H36C	109.5
C40—N8—H8n	115.2	H36A—C36—H36C	109.5
C47—N9—C48	115.2 (2)	H36B—C36—H36C	109.5
C50—N10—C47	116.3 (2)	C38—C37—C35	111.0 (3)
C50—N12—C63	129.9 (2)	C38—C37—C36	111.4 (3)
C50—N12—H12n	115.0	C35—C37—C36	110.0 (2)
C63—N12—H12n	115.0	C38—C37—H37	108.1
N1—C1—N2	129.3 (2)	C35—C37—H37	108.1
N1—C1—S1	119.08 (18)	C36—C37—H37	108.1
N2—C1—S1	111.62 (18)	C37—C38—H38A	109.5
N1—C2—C3	120.4 (2)	C37—C38—H38B	109.5
N1—C2—C12	116.7 (2)	H38A—C38—H38B	109.5
C3—C2—C12	122.9 (2)	C37—C38—H38C	109.5
C2—C3—C4	119.0 (2)	H38A—C38—H38C	109.5
C2—C3—C16	120.6 (2)	H38B—C38—H38C	109.5
C4—C3—C16	120.4 (2)	N7—C39—C26	177.3 (3)
N2—C4—N4	119.5 (2)	C45—C40—C41	119.5 (2)
N2—C4—C3	119.6 (2)	C45—C40—N8	116.2 (2)
N4—C4—C3	121.0 (2)	C41—C40—N8	124.3 (2)
C6—C5—S1	114.73 (17)	C42—C41—C40	119.6 (2)
C6—C5—H5A	108.6	C42—C41—H41	120.2
S1—C5—H5A	108.6	C40—C41—H41	120.2
C6—C5—H5B	108.6	C41—C42—C43	121.2 (3)
S1—C5—H5B	108.6	C41—C42—H42	119.4
H5A—C5—H5B	107.6	C43—C42—H42	119.4
C7—C6—C11	118.4 (2)	C42—C43—C44	118.5 (3)
C7—C6—C5	119.0 (2)	C42—C43—H43	120.7
C11—C6—C5	122.5 (2)	C44—C43—H43	120.7
C8—C7—C6	121.3 (3)	C45—C44—C43	121.0 (2)
C8—C7—H7	119.3	C45—C44—C46	118.5 (2)
C6—C7—H7	119.3	C43—C44—C46	120.4 (2)
C9—C8—C7	118.7 (2)	C44—C45—C40	120.1 (2)
C9—C8—H8A	120.7	C44—C45—H45	120.0
C7—C8—H8A	120.7	C40—C45—H45	120.0
C8—C9—C10	121.8 (3)	F4—C46—F6	105.8 (2)
C8—C9—Cl1	119.0 (2)	F4—C46—F5	105.9 (2)
C10—C9—Cl1	119.2 (2)	F6—C46—F5	106.2 (2)
C9—C10—C11	118.5 (3)	F4—C46—C44	112.5 (2)
C9—C10—H10	120.8	F6—C46—C44	112.5 (2)
C11—C10—H10	120.8	F5—C46—C44	113.2 (2)
C10—C11—C6	121.3 (2)	N9—C47—N10	129.1 (2)
C10—C11—H11	119.3	N9—C47—S3	119.56 (18)
C6—C11—H11	119.3	N10—C47—S3	111.38 (18)
C2—C12—C14	114.1 (2)	N9—C48—C49	120.7 (2)
C2—C12—H12A	108.7	N9—C48—C58	117.5 (2)
C14—C12—H12A	108.7	C49—C48—C58	121.7 (2)
C2—C12—H12B	108.7	C48—C49—C50	119.2 (2)
C14—C12—H12B	108.7	C48—C49—C62	120.9 (2)
H12A—C12—H12B	107.6	C50—C49—C62	119.9 (2)
C14—C13—H13A	109.5	N10—C50—N12	120.3 (2)
C14—C13—H13B	109.5	N10—C50—C49	119.4 (2)
H13A—C13—H13B	109.5	N12—C50—C49	120.3 (2)
C14—C13—H13C	109.5	C52—C51—S3	115.19 (18)
H13A—C13—H13C	109.5	C52—C51—H51A	108.5
H13B—C13—H13C	109.5	S3—C51—H51A	108.5
C13—C14—C15	111.8 (3)	C52—C51—H51B	108.5
C13—C14—C12	114.2 (3)	S3—C51—H51B	108.5
C15—C14—C12	111.5 (2)	H51A—C51—H51B	107.5
C13—C14—H14	106.2	C53—C52—C57	119.0 (2)
C15—C14—H14	106.2	C53—C52—C51	120.0 (2)
C12—C14—H14	106.2	C57—C52—C51	121.0 (2)
C14—C15—H15A	109.5	C52—C53—C54	120.8 (3)
C14—C15—H15B	109.5	C52—C53—H53	119.6
H15A—C15—H15B	109.5	C54—C53—H53	119.6
C14—C15—H15C	109.5	C55—C54—C53	118.9 (3)
H15A—C15—H15C	109.5	C55—C54—H54	120.6
H15B—C15—H15C	109.5	C53—C54—H54	120.6
N3—C16—C3	178.5 (3)	C54—C55—C56	122.0 (3)
C22—C17—C18	119.2 (2)	C54—C55—Cl3	119.4 (2)
C22—C17—N4	115.5 (2)	C56—C55—Cl3	118.6 (2)
C18—C17—N4	125.3 (2)	C55—C56—C57	118.4 (3)
C19—C18—C17	119.2 (3)	C55—C56—H56	120.8
C19—C18—H18	120.4	C57—C56—H56	120.8
C17—C18—H18	120.4	C52—C57—C56	120.9 (2)
C20—C19—C18	122.2 (3)	C52—C57—H57	119.5
C20—C19—H19	118.9	C56—C57—H57	119.5
C18—C19—H19	118.9	C48—C58—C60	114.7 (2)
C19—C20—C21	118.0 (3)	C48—C58—H58A	108.6
C19—C20—H20	121.0	C60—C58—H58A	108.6
C21—C20—H20	121.0	C48—C58—H58B	108.6
C22—C21—C20	120.8 (2)	C60—C58—H58B	108.6
C22—C21—C23	119.1 (3)	H58A—C58—H58B	107.6
C20—C21—C23	120.0 (3)	C60—C59—H59A	109.5
C21—C22—C17	120.5 (2)	C60—C59—H59B	109.5
C21—C22—H22	119.7	H59A—C59—H59B	109.5
C17—C22—H22	119.7	C60—C59—H59C	109.5
F2—C23—F1	107.0 (3)	H59A—C59—H59C	109.5
F2—C23—F3	105.3 (3)	H59B—C59—H59C	109.5
F1—C23—F3	105.3 (3)	C61—C60—C59	111.2 (2)
F2—C23—C21	113.4 (3)	C61—C60—C58	112.2 (2)
F1—C23—C21	112.4 (3)	C59—C60—C58	108.9 (2)
F3—C23—C21	112.8 (3)	C61—C60—H60	108.1
N5—C24—N6	129.1 (2)	C59—C60—H60	108.1
N5—C24—S2	119.42 (19)	C58—C60—H60	108.1
N6—C24—S2	111.45 (19)	C60—C61—H61A	109.5
N5—C25—C26	120.8 (2)	C60—C61—H61B	109.5
N5—C25—C35	116.2 (2)	H61A—C61—H61B	109.5
C26—C25—C35	123.1 (2)	C60—C61—H61C	109.5
C25—C26—C27	119.1 (2)	H61A—C61—H61C	109.5
C25—C26—C39	121.3 (2)	H61B—C61—H61C	109.5
C27—C26—C39	119.5 (2)	N11—C62—C49	177.7 (3)
N6—C27—N8	120.0 (2)	C64—C63—C68	119.2 (2)
N6—C27—C26	119.3 (2)	C64—C63—N12	124.5 (2)
N8—C27—C26	120.7 (2)	C68—C63—N12	116.3 (2)
C29—C28—S2	114.89 (18)	C65—C64—C63	119.8 (2)
C29—C28—H28A	108.5	C65—C64—H64	120.1
S2—C28—H28A	108.5	C63—C64—H64	120.1
C29—C28—H28B	108.5	C64—C65—C66	121.4 (2)
S2—C28—H28B	108.5	C64—C65—H65	119.3
H28A—C28—H28B	107.5	C66—C65—H65	119.3
C30—C29—C34	118.4 (3)	C67—C66—C65	118.4 (2)
C30—C29—C28	119.3 (2)	C67—C66—H66	120.8
C34—C29—C28	122.3 (2)	C65—C66—H66	120.8
C31—C30—C29	121.6 (3)	C66—C67—C68	121.0 (2)
C31—C30—H30	119.2	C66—C67—C69	120.3 (2)
C29—C30—H30	119.2	C68—C67—C69	118.7 (2)
C32—C31—C30	118.9 (3)	C67—C68—C63	120.1 (2)
C32—C31—H31	120.5	C67—C68—H68	119.9
C30—C31—H31	120.5	C63—C68—H68	119.9
C31—C32—C33	121.6 (3)	F7—C69—F8	106.7 (3)
C31—C32—Cl2	119.0 (2)	F7—C69—F9	105.5 (3)
C33—C32—Cl2	119.4 (2)	F8—C69—F9	105.2 (3)
C32—C33—C34	118.5 (2)	F7—C69—C67	113.0 (3)
C32—C33—H33	120.8	F8—C69—C67	113.0 (2)
C34—C33—H33	120.8	F9—C69—C67	112.9 (2)
C29—C34—C33	121.0 (2)		
			
C2—N1—C1—N2	0.7 (4)	C31—C32—C33—C34	2.9 (4)
C2—N1—C1—S1	179.89 (17)	Cl2—C32—C33—C34	−177.75 (18)
C4—N2—C1—N1	0.2 (4)	C30—C29—C34—C33	−1.7 (4)
C4—N2—C1—S1	−179.04 (17)	C28—C29—C34—C33	177.8 (2)
C5—S1—C1—N1	12.8 (2)	C32—C33—C34—C29	−1.1 (4)
C5—S1—C1—N2	−167.86 (17)	N5—C25—C35—C37	−68.5 (3)
C1—N1—C2—C3	−1.8 (3)	C26—C25—C35—C37	111.9 (3)
C1—N1—C2—C12	178.1 (2)	C25—C35—C37—C38	−65.3 (3)
N1—C2—C3—C4	2.0 (3)	C25—C35—C37—C36	170.9 (3)
C12—C2—C3—C4	−177.9 (2)	C27—N8—C40—C45	−154.6 (2)
N1—C2—C3—C16	−178.0 (2)	C27—N8—C40—C41	27.1 (4)
C12—C2—C3—C16	2.1 (4)	C45—C40—C41—C42	1.0 (4)
C1—N2—C4—N4	−179.2 (2)	N8—C40—C41—C42	179.2 (3)
C1—N2—C4—C3	0.0 (3)	C40—C41—C42—C43	−0.7 (4)
C17—N4—C4—N2	1.9 (4)	C41—C42—C43—C44	0.1 (5)
C17—N4—C4—C3	−177.3 (2)	C42—C43—C44—C45	0.2 (4)
C2—C3—C4—N2	−1.0 (3)	C42—C43—C44—C46	177.3 (3)
C16—C3—C4—N2	179.0 (2)	C43—C44—C45—C40	0.1 (4)
C2—C3—C4—N4	178.2 (2)	C46—C44—C45—C40	−177.0 (2)
C16—C3—C4—N4	−1.8 (4)	C41—C40—C45—C44	−0.7 (4)
C1—S1—C5—C6	67.3 (2)	N8—C40—C45—C44	−179.1 (2)
S1—C5—C6—C7	−147.4 (2)	C45—C44—C46—F4	80.5 (3)
S1—C5—C6—C11	32.8 (3)	C43—C44—C46—F4	−96.6 (3)
C11—C6—C7—C8	−0.4 (4)	C45—C44—C46—F6	−38.9 (4)
C5—C6—C7—C8	179.8 (2)	C43—C44—C46—F6	143.9 (3)
C6—C7—C8—C9	0.0 (4)	C45—C44—C46—F5	−159.4 (2)
C7—C8—C9—C10	0.6 (4)	C43—C44—C46—F5	23.4 (4)
C7—C8—C9—Cl1	179.5 (2)	C48—N9—C47—N10	1.4 (4)
C8—C9—C10—C11	−0.7 (4)	C48—N9—C47—S3	179.95 (17)
Cl1—C9—C10—C11	−179.6 (2)	C50—N10—C47—N9	0.1 (4)
C9—C10—C11—C6	0.3 (4)	C50—N10—C47—S3	−178.53 (16)
C7—C6—C11—C10	0.2 (4)	C51—S3—C47—N9	4.5 (2)
C5—C6—C11—C10	−180.0 (2)	C51—S3—C47—N10	−176.72 (17)
N1—C2—C12—C14	−55.0 (3)	C47—N9—C48—C49	−2.4 (3)
C3—C2—C12—C14	124.8 (3)	C47—N9—C48—C58	178.7 (2)
C2—C12—C14—C13	−50.7 (4)	N9—C48—C49—C50	2.0 (4)
C2—C12—C14—C15	−178.7 (2)	C58—C48—C49—C50	−179.1 (2)
C4—N4—C17—C22	167.8 (2)	N9—C48—C49—C62	−176.8 (2)
C4—N4—C17—C18	−12.9 (4)	C58—C48—C49—C62	2.1 (4)
C22—C17—C18—C19	−1.6 (4)	C47—N10—C50—N12	179.8 (2)
N4—C17—C18—C19	179.1 (3)	C47—N10—C50—C49	−0.6 (3)
C17—C18—C19—C20	1.0 (5)	C63—N12—C50—N10	−2.6 (4)
C18—C19—C20—C21	0.1 (5)	C63—N12—C50—C49	177.8 (2)
C19—C20—C21—C22	−0.4 (5)	C48—C49—C50—N10	−0.4 (3)
C19—C20—C21—C23	175.9 (3)	C62—C49—C50—N10	178.4 (2)
C20—C21—C22—C17	−0.3 (4)	C48—C49—C50—N12	179.2 (2)
C23—C21—C22—C17	−176.7 (3)	C62—C49—C50—N12	−2.0 (3)
C18—C17—C22—C21	1.3 (4)	C47—S3—C51—C52	74.5 (2)
N4—C17—C22—C21	−179.4 (2)	S3—C51—C52—C53	−138.2 (2)
C22—C21—C23—F2	−152.7 (3)	S3—C51—C52—C57	42.9 (3)
C20—C21—C23—F2	30.9 (4)	C57—C52—C53—C54	−0.5 (4)
C22—C21—C23—F1	85.8 (4)	C51—C52—C53—C54	−179.5 (2)
C20—C21—C23—F1	−90.6 (4)	C52—C53—C54—C55	−0.7 (4)
C22—C21—C23—F3	−33.1 (4)	C53—C54—C55—C56	1.6 (4)
C20—C21—C23—F3	150.5 (3)	C53—C54—C55—Cl3	−178.3 (2)
C25—N5—C24—N6	2.4 (4)	C54—C55—C56—C57	−1.3 (4)
C25—N5—C24—S2	−178.31 (17)	Cl3—C55—C56—C57	178.60 (19)
C27—N6—C24—N5	−3.0 (4)	C53—C52—C57—C56	0.9 (4)
C27—N6—C24—S2	177.71 (17)	C51—C52—C57—C56	179.8 (2)
C28—S2—C24—N5	11.2 (2)	C55—C56—C57—C52	0.0 (4)
C28—S2—C24—N6	−169.36 (18)	N9—C48—C58—C60	−44.6 (3)
C24—N5—C25—C26	1.4 (3)	C49—C48—C58—C60	136.5 (3)
C24—N5—C25—C35	−178.2 (2)	C48—C58—C60—C61	−54.3 (3)
N5—C25—C26—C27	−4.2 (4)	C48—C58—C60—C59	−177.9 (2)
C35—C25—C26—C27	175.4 (2)	C50—N12—C63—C64	16.2 (4)
N5—C25—C26—C39	177.9 (2)	C50—N12—C63—C68	−165.5 (2)
C35—C25—C26—C39	−2.5 (4)	C68—C63—C64—C65	0.8 (4)
C24—N6—C27—N8	179.9 (2)	N12—C63—C64—C65	179.1 (2)
C24—N6—C27—C26	−0.3 (3)	C63—C64—C65—C66	−0.4 (4)
C40—N8—C27—N6	−6.2 (4)	C64—C65—C66—C67	−0.6 (4)
C40—N8—C27—C26	174.0 (2)	C65—C66—C67—C68	1.2 (4)
C25—C26—C27—N6	3.6 (3)	C65—C66—C67—C69	179.7 (3)
C39—C26—C27—N6	−178.5 (2)	C66—C67—C68—C63	−0.7 (4)
C25—C26—C27—N8	−176.5 (2)	C69—C67—C68—C63	−179.3 (2)
C39—C26—C27—N8	1.3 (4)	C64—C63—C68—C67	−0.3 (4)
C24—S2—C28—C29	71.8 (2)	N12—C63—C68—C67	−178.8 (2)
S2—C28—C29—C30	−126.3 (2)	C66—C67—C69—F7	−104.6 (3)
S2—C28—C29—C34	54.1 (3)	C68—C67—C69—F7	74.0 (4)
C34—C29—C30—C31	3.0 (4)	C66—C67—C69—F8	16.6 (4)
C28—C29—C30—C31	−176.6 (3)	C68—C67—C69—F8	−164.8 (3)
C29—C30—C31—C32	−1.3 (5)	C66—C67—C69—F9	135.8 (3)
C30—C31—C32—C33	−1.7 (4)	C68—C67—C69—F9	−45.6 (4)
C30—C31—C32—Cl2	178.9 (2)		

### Hydrogen-bond geometry (Å, º)

**Table:**

*D*—H···*A*	*D*—H	H···*A*	*D*···*A*	*D*—H···*A*
N4—H4*n*···N7^i^	0.88	2.39	3.227 (3)	159
N8—H8*n*···N3^i^	0.88	2.38	3.213 (3)	158
N12—H12*n*···N11^ii^	0.88	2.36	3.206 (3)	160

Symmetry codes: (i) −*x*+1, −*y*+1, −*z*; (ii) −*x*+2, −*y*+1, −*z*.
